# Performance Evaluation of a Magnetically Driven Microrobot for Targeted Drug Delivery

**DOI:** 10.3390/mi12101210

**Published:** 2021-10-03

**Authors:** Zhuocong Cai, Qiang Fu, Songyuan Zhang, Chunliu Fan, Xi Zhang, Jian Guo, Shuxiang Guo

**Affiliations:** 1 Intelligent Robot Laboratory, Tianjin University of Technology, Tianjin 300380, China; caizhuocong@hotmail.com (Z.C.); fanchunliu2536723678@hotmail.com (C.F.); zhangxi549244209@hotmail.com (X.Z.); 2 Tianjin Key Laboratory for Control Theory & Application in Complicated Systems and Tianjin International Joint Research and Development Center, Tianjin University of Technology, Tianjin 300380, China; gj15102231710@163.com; 3 State Key Laboratory of Robotics and System, Harbin Institute of Technology, Harbin 150001, China; 4 Department of Intelligent Mechanical Systems Engineering, Kagawa University, Takamatsu 761-0396, Japan; guo@eng.kagawa-u.ac.jp

**Keywords:** targeted drug delivery microrobot, cam structure, magnetic actuated capsule microrobotic system (MACMS), Computational Fluid Dynamics (CFD)

## Abstract

Given that the current microrobot cannot achieve fixed-point and quantitative drug application in the gastrointestinal (GI) tract, a targeted drug delivery microrobot is proposed, and its principle and characteristics are studied. Through the control of an external magnetic field, it can actively move to the affected area to realize the targeted drug delivery function. The microrobot has a cam structure connected with a radially magnetized permanent magnet, which can realize two movement modes: movement and targeted drug delivery. Firstly, the magnetic actuated capsule microrobotic system (MACMS) is analyzed. Secondly, the dynamic model and quantitative drug delivery model of the targeted drug delivery microrobot driven by the spiral jet structure are established, and the motion characteristics of the targeted drug delivery microrobot are simulated and analyzed by the method of Computational Fluid Dynamics (CFD). Finally, the whole process of the targeted drug delivery task of the microrobot is simulated. The results show that the targeted drug delivery microrobot can realize basic movements such as forward, backward, fixed-point parking and drug delivery through external magnetic field control, which lays the foundation for gastrointestinal diagnosis and treatment.

## 1. Introduction

Microrobots have been widely studied because of their promising application in GI diagnosis and treatment [1,2,3]. Compared with the traditional electronic endoscope, the microrobot has great research potential due to its safety, reliability and painless effect [4,5,6]. Although the microrobot has been used to carry out motion and image experiments in the pipeline or animal GI tract, it is far from sufficient to only have an image function in the clinic. When some GI diseases need to be treated with drugs, the use of surgical methods will cause great damage to patients. Meanwhile, oral drug administration means that the drugs are easily affected by the GI tract or gastric acid, so it is difficult to improve the drug concentration at the focus and reduce the treatment effect [7]. Realizing the drug delivery function of a microrobot in the human GI has become a research problem.

In order to solve this problem, many researchers have carried out relevant research. Koga et al. proposed a microrobot with pasting and drug injection functions. This microrobot is driven by air pressure, and an air sac on its surface expands and pushes its surface toward the inner wall of the intestine to drive an integrated ejector to release drugs [8]. Beccani et al. proposed a magnetic drug delivery microrobot. The drug delivery device of the microrobot is based on a coil driving mechanism, which can be anchored in one position and releases all drugs by a wireless device [9]. Guo et al. proposed a microrobot for a drug release system; its drug release mechanism is composed of a one-way drug release valve, a drug chamber, two axially magnetized cylindrical permanent magnets and a multilayer solenoid coil. In addition, the executive force is controlled by adjusting the activation cycle and intensity of the coil, to release the drug in the GI tract [10].

However, if the movement to the lesion site is not precise and only a large area of application function is considered, the normal tissue will be affected and the lesion site will not be well treated [11,12,13]. Yim et al. proposed a magnetically driven soft microrobot that is remotely driven by two embedded permanent magnets and a large external magnet. The external attraction force is used to anchor the microrobot in the desired position and release drugs by squeezing the body of the microrobot [14]. Kim et al. proposed a microrobot that could move actively and release targeted drugs. The external magnetic field made the microrobot produce two rolling motions and one rotating motion for active motion. When the microrobot arrived at the destination, it could release drugs by switching the thrust direction of the two spiral components [15]. Le et al. proposed a drug delivery microrobot in which the drug delivery module is driven by a precisely controlled external magnetic field. The drug delivery module is opened by repulsion between two radially magnetized soft magnetic rings, and the encapsulated drug can be released to achieve drug treatment at the lesion location [16].

However, these studies did not provide a detailed analysis of the targeted drug application process. In addition, the coupling problem of multiple magnets to release drugs in the magnetic field makes doctors unable to accurately locate the microrobot, and this affects the treatment of the lesion [17,18,19]. Therefore, we propose a novel targeted drug delivery microrobot, which can not only actively move to the lesion position controlled by the external magnetic field, but also quantitatively control the drug release through a drug application mechanism fixed by the internal single permanent magnet and cam structure. The single permanent magnet is used to realize the two functions of motion and drug application, to realize the function of targeted drug application.

This paper is structured as follows. Firstly, the MACMS system is introduced. Secondly, the structure and functional principle, dynamic model and quantitative drug delivery model of the targeted drug delivery microrobot are discussed. The fourth part analyzes the drug delivery process of the targeted drug delivery microrobot, and the kinematics model of drug delivery is established; then, the external magnetic field is analyzed by electromagnetic simulation, and then the drug delivery process is analyzed by hydrodynamics simulation. The fifth part evaluates the motion performance and drug delivery control conditions of the targeted drug delivery microrobot, and the experimental results are analyzed. Finally, the conclusions and future work are given.

## 2. Electromagnetic System Configuration of Targeted Drug Delivery Microrobot

**Figure 1:**
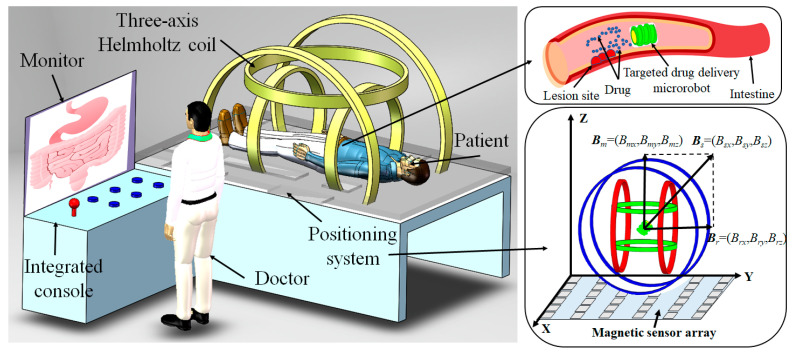
Electromagnetic system configuration of targeted drug delivery microrobot.

As shown in Figure 1, MACMS is divided into a master control system and slave treatment system [11]. The operator controls the whole system through the main control system, and directly controls the targeted drug delivery microrobot from the slave treatment system to carry out the GI-targeted drug delivery task. The principle of the system is to generate a universal space rotating magnetic field by controlling the three-axis Helmholtz coil to control the movement, attitude adjustment and targeted drug delivery of the targeted drug delivery microrobot driven by a spiral jet through the current driving method [20].

In order to accurately control the rotation direction and torque of the permanent magnet embedded in the targeted drug delivery microrobot, a three-axis Helmholtz coil is used for the purpose of providing energy, the rotation speed of the microrobot is controlled by controlling the frequency of the magnetic field, and the magnetic torque is controlled by controlling the magnetic induction intensity of the magnetic field [21]. The radius and number of turns of the three axes are *R_x_* = 293.4 mm, *R_y_* = 211.4 mm, *R_z_* = 157.4 mm, *N_x_* = 216, *N_y_* = 174, *N_z_* = 126, respectively. The magnetic induction intensity range after current is applied is 0–30 Gs.

The positioning system is composed of a 6 × 6 magnetic sensor array, as shown in Figure 1. By selecting the *x*, *y*, *z*, rolling, pitch and yaw parameters of the targeted drug delivery microrobot as the output of the position parameters, the position of the microrobot is solved by the least square method, and the problem of magnetic coupling is solved by the magnetic positioning compensation algorithm [12]. The positioning error of the microrobot is fitted three times to make the positioning accuracy reach 2.25 mm. The positioning system can enable the operator to grasp the position and attitude of the targeted drug delivery microrobot more accurately in the human body, which is convenient for the implementation of the targeted drug application task.

By setting the rotation speed and rotation direction of the external rotating magnetic field, the movement direction and speed of the targeted drug delivery microrobot can be controlled in real time, so that the microrobot can accurately reach the affected area and carry out the drug application work, as shown in Figure 1. After the task is completed, it will be discharged with the waste. The targeted drug delivery microrobot controlled by MACMS can realize active and accurate drug delivery, effectively reduce patients’ pain and GI tissue injury and promote patients’ rehabilitation.

## 3. Targeted Drug Delivery Microrobot

### 3.1. Motion Mechanism of Targeted Drug Delivery Microrobot

**Figure 2:**
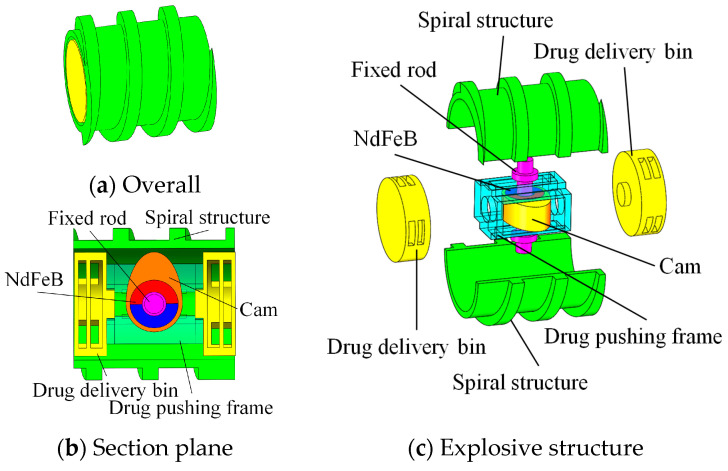
Structure of targeted drug delivery microrobot.

The model of the targeted drug delivery microrobot is shown in Figure 2a. Since the spiral jet structure can provide a stable propulsion force for the microrobot, the shell adopts a spiral jet structure. The rotation of the spiral jet structure drives the fluid to rotate, and the reaction force provided by the fluid can make the targeted microrobot advance. If the spiral jet structure rotates in the opposite direction, the microrobot can move in the opposite direction.

The structure of the targeted drug delivery microrobot is shown in Figure 2b, which consists of a fixed rod, a drug pushing frame, a cam, a NdFeB (neodymium iron boron) permanent magnet and a drug delivery bin. The cam and permanent magnet are fixed, and the protruding part of the cam is magnetized in the same direction as the radial permanent magnet. Because a drug cannot meet the treatment requirements of some diseases, the drug delivery bin at each end can be divided into groups carrying multiple drugs. The drug delivery bin, pushing frame, cam and permanent magnet are consolidated into a targeted application device. When the permanent magnet inside the targeted drug delivery microrobot rotates axially, the microrobot can rotate and move; when the permanent magnet rotates radially, the cam is driven by the permanent magnet for targeted drug application. It can realize one input signal and multiple output modes.

Using a light-curing 3D printer, medical photosensitive resin was used to print the targeted drug delivery microrobot. The main structural parameters of the targeted drug delivery microrobot are shown in Table 1. The structural dimensions of the design meet both process requirements and medical dimensional requirements.

**Figure 3:**
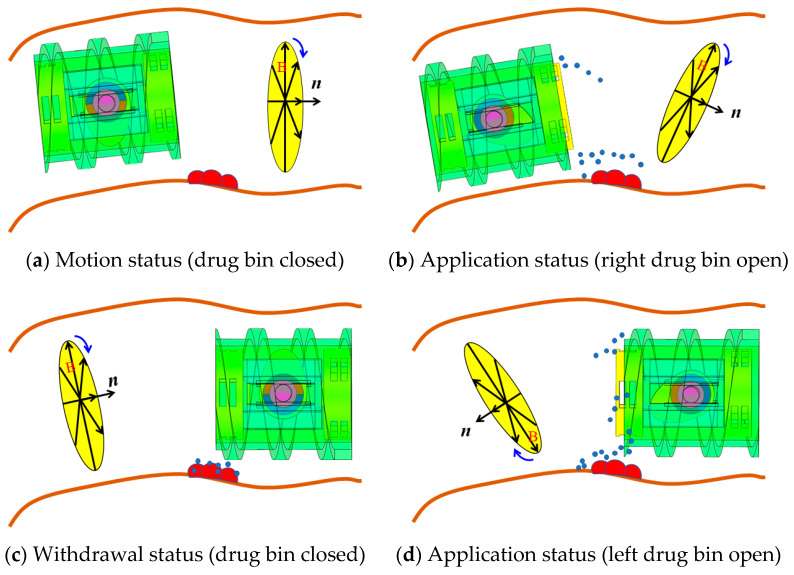
Motion mechanism of targeted drug delivery microrobot.

The motion mechanism of the targeted drug delivery microrobot is shown in Figure 3. The permanent magnet rotates with the rotation of the magnetic field. The microrobot advances due to the reaction force of the fluid due to the rotation of the spiral structure. At this time, the state is as shown in Figure 3a. When the drug needs to be applied to the affected area, the state of the targeted drug delivery microrobot is as shown in Figure 3b. The magnetic field in any direction can be superimposed by controlling the external magnetic field. The rotation of the permanent magnet drives the cam to rotate and pushes the drug bin to apply the drug to the affected area. The drug amount is determined by accurately controlling the rotation angle of the cam. After the drug is released, the direction of the magnetic field is changed, the drug pushing frame is pushed through the cam, and the drug bin that has released the drug is pulled back. At this time, the state of the targeted drug delivery microrobot is as shown in Figure 3c. When another drug is needed, change the direction of the magnetic field, and push another drug bin to release the drug, as shown in Figure 3d. After the targeted drug application task is completed, the targeted drug delivery microrobot returns to the motion state and is finally discharged from the body.

### 3.2. Dynamic Model

**Figure 4:**
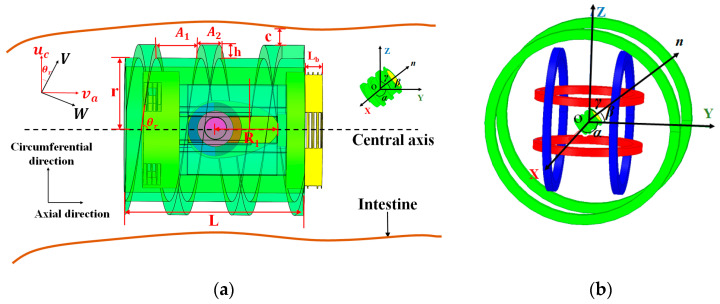
(**a**) Dynamic model of targeted drug delivery microrobot. (**b**) Magnetic vector analysis model.

In order to analyze the motion characteristics of the targeted drug delivery microrobot, the microrobot dynamic model is established based on hydrodynamics theory [22], as shown in Figure 4a.

For the convenience of analysis, the circumferential velocity *u_c_* and axial velocity *v_a_* of the targeted drug delivery microrobot during rotation are decomposed along the spiral direction and perpendicular to the spiral direction, respectively, to form the velocity *W* along the spiral direction and the velocity *V* perpendicular to the spiral direction [23]. *θ_r_* is the spiral rise angle of the helix, *a* is the pitch, *b* is the thread width, *h* is the thread height, *c* is the gap between the top of the thread and the inner wall of the intestinal tract, and *μ* is the fluid viscosity. Then, *W* and *V* can be obtained from *u_c_* and *v_a_*:

(1) $$\left[\begin{array}{c} W\\ V \end{array}\right]=\left[\begin{array}{cc} \cos\theta_{r} & -\sin\theta_{r}\\ \cos\theta_{r} & \sin\theta_{r} \end{array}\right]\left[\begin{array}{c} v_{a}\\ u_{c} \end{array}\right]$$

In order to facilitate the calculation, pressure analysis is carried out only within one pitch range of the outer surface of microrobot. Since the pressure in the *Y* direction does not change, i.e., *dP*/*dy* = 0, according to the Reynolds equation, it can be obtained [24]:

(2) $$\frac{d}{dx}\left(h^{3}\frac{dP}{dx}\right)=6\mu w\frac{dh}{dx}$$

where *P* is the dynamic pressure of the fluid between the spiral surface and the pipe wall, and *μ* is the viscosity of the fluid. After integral operation, *P* can be expressed as:

(3) $$P=6\mu w\frac{h-h_{0}}{h^{3}}x+c_{0}$$

where *h*_0_ and *c*_0_ are undetermined coefficients, which are determined as *h* and *c* in the *A_1_* and *A*_2_ regions. *P*_1_ and *P*_2_ are obtained by bringing the parameters of the *A*_1_ and *A*_2_ regions into Equation (3).

According to the Reynolds equation, the pressure distribution on a thread cycle can be obtained:

(4) $$P_{1}\left(x\right)=\frac{6\mu \gamma \left(1-\beta_{r}\right)W_{1}{}^{2}x}{c^{2}{\left(1+\gamma_{r}\right)}^{2}\left[\left(1-\beta_{r}\right)W_{1}+\beta_{r}{\left(1+\gamma_{r}\right)}^{3}W_{2}\right]}$$

(5) $$P_{2}\left(x\right)=-\frac{\left[6\mu \beta_{r}\gamma_{r}{\left(1+\gamma_{r}\right)}^{2}W_{2}{}^{2}\right]\left(A_{1}+A_{2}-x\right)}{c^{2}\left[\left(1-\beta_{r}\right)W_{1}+\beta_{r}{\left(1+\gamma_{r}\right)}^{3}W_{2}\right]}$$

where *β_r_* = *b*/(*a* + *b*), *γ_r_* = *h*/*c*.

According to Newton’s law of internal friction, the shear stress of the fluid when the threaded structure rotates can be obtained:

(6) $$F_{x}=\frac{2\pi L\mu }{c}\{\frac{3W_{1}W_{2}\beta_{r}\gamma_{r}\left[\left(r-c\gamma_{r}\right)\left(1-\beta_{r}\right)\left(1+\gamma_{r}\right)-r\right]}{\left(1-\beta_{r}\right)W_{1}+\beta_{r}{\left(1+\gamma_{r}\right)}^{3}W_{2}}-\frac{W_{1}\left(r-c\gamma_{r}\right)\left(1-\beta_{r}\right)}{1+\gamma_{r}}-r\beta_{r}W_{2}\}$$

(7) $$F_{y}=-\frac{2\pi L\mu }{c}\left[\frac{\left(r-c\gamma_{r}\right)\left(1-\beta_{r}\right)}{1+\gamma_{r}}V_{1}+r\beta_{r}V_{2}\right]$$

Therefore, the propulsive force *F_p_* and the resistance *F_r_* when the targeted drug delivery microrobot moves is expressed as:

(8) $$\{\begin{array}{c} F_{p}=F_{y}\sin\theta_{r}-F_{x}\cos\theta_{r}\\ F_{r}=F_{y}\cos\theta_{r}+F_{x}\sin\theta_{r} \end{array}$$

### 3.3. Quantitative Targeted Delivery Model

In order to control the targeted drug delivery microrobot to accurately deliver drugs to the affected area, Cartesian coordinates are established in the three-axis Helmholtz coil. As shown in Figure 4b, the targeted drug delivery microrobot locates in the uniform magnetic field generated by the three-axis Helmholtz coil, the coordinate system (OXYZ) is consolidated in its center, and ***n*** is the normal vector of the rotating magnetic field plane; *α*, *β* and *γ* are the included angles between ***n*** and the *X* axis, *Y* axis and *Z* axis, respectively, and ***n*** can be expressed as (cos*α*, cos*β*, cos*γ*). The current driving method of the targeted drug delivery microrobot in the three-axis Helmholtz coil can be as follows [24]:

(9) $$\{\begin{array}{c} I_{x}=-I_{0}\sin(\alpha )\sin\left(\omega t-\varphi_{x}\right)\\ I_{y}=-I_{0}\sin(\beta )\sin\left(\omega t+\varphi_{y}\right)\\ I_{z}=I_{0}\sin(\gamma )\sin\left(\omega t+\pi /2\right) \end{array}$$

where tan*φ_x_* = (cos*β/*cos*α*·cos*γ*), tan*φ_y_* = (cos*α/*cos*β*·cos*γ*), *I_x_*, *I_y_* and *I_z_* are the amplitude of the input currents of the three coils, respectively, *I_0_* is the current amplitude, and *ω* is the angular frequency of the current.

**Figure 5:**
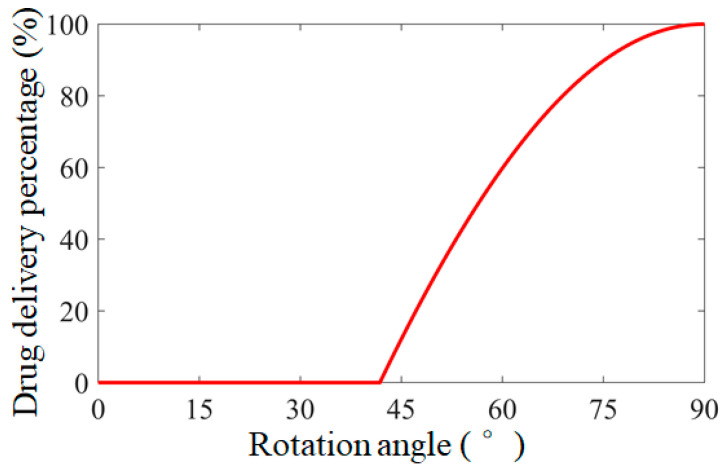
The relationship between percentage of drug delivery bin and the rotation angle of the permanent magnet in the targeted drug delivery microrobot.

In order to deal with various diseases, the pushing rate of the targeted drug delivery microrobot needs to be accurately calculated. After the cam in the targeted drug delivery microrobot is driven by the permanent magnet to rotate, the normal vector of the magnetic field surface changes from ***n*** to ***n*_1_**, and the rotation angle is *θ*, which is the angle between the vectors ***n*** and ***n*_1_**, so the percentage of drug delivery bin *η* can be expressed as:

(10) $$\eta =\frac{R_{1}\sin\theta +L_{b}-\frac{1}{2}L}{R_{1}+L_{b}-\frac{1}{2}L}\times 100\%$$

where *R*_1_ is the long radius of the cam structure and *L_b_* is the length of the drug bin. The targeted drug delivery microrobot can accurately control the percentage of drug delivery bin by controlling the radial rotation angle of the permanent magnet. The relationship between the rotation angle of the permanent magnet consolidated cam structure of the targeted drug delivery microrobot and the percentage of the drug delivery bin is shown in Figure 5. According to the structural parameters of the targeted drug delivery microrobot, it can be obtained that the drug delivery starts when the permanent magnet rotates to 41°. When the permanent magnet rotates to 90°, the percentage of the drug delivery bin is 100% and all drugs can be released. The type and quantity of drugs can be determined according to the condition of the lesion, and the targeted drug delivery microrobot can be controlled to move to any position in the human intestine through the external magnetic field to achieve an accurate targeted drug delivery function.

## 4. Analysis of Drug Delivery Process of Targeted Drug Delivery Microrobot

### 4.1. Model of Drug Delivery

According to Newton’s second law, the kinematics model of the targeted drug delivery microrobot in the process of drug application is analyzed, and the force distribution of the microrobot is simplified, including the resultant force *F_rb_*, the propulsive force *F_pb_* of the cam pushing the drug bin and the resistance *F_reb_* of the fluid when the drug bin goes out, and their relationship can be expressed as:

(11) $$F_{rb}=F_{pb}-F_{reb}$$

where the propulsive force *F_pb_* of the cam pushing the drug bin and the resistance *F_reb_* of the fluid when the drug bin goes out can be expressed as:

(12) $$F_{pb}=\frac{T\cdot \cos\theta }{R_{1}}$$

(13) $$F_{reb}=0.5C_{re}\cdot \rho \cdot S\cdot v_{b}{}^{2}$$

where *T_m_* is the magnetic torque, *ρ* is the density of the fluid, *S* is the maximum cross-sectional area perpendicular to the fluid flow, *v_b_* is the speed of the drug bin of the targeted delivery microrobot, and *C_re_* is the resistance coefficient.

### 4.2. Electromagnetic Analysis

When the permanent magnet inside the targeted drug delivery microrobot is rotating, the torque *T_m_* produced by magnetic field can be given as:

(14) $$T_{m}=Bm\sin\theta_{v}$$

where *B* is the magnetic induction intensity, *m* is the magnetic moment vector of the NdFeB permanent magnet inside the targeted drug delivery microrobot, and *θ_v_* is the angle between the rotating magnetic field vector and the magnetic moment vector of the NdFeB permanent magnet.

**Figure 6:**
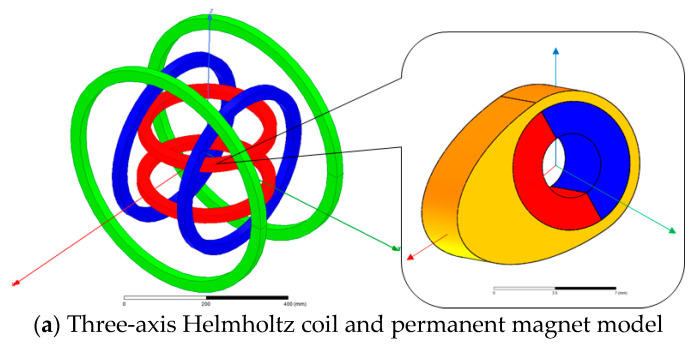

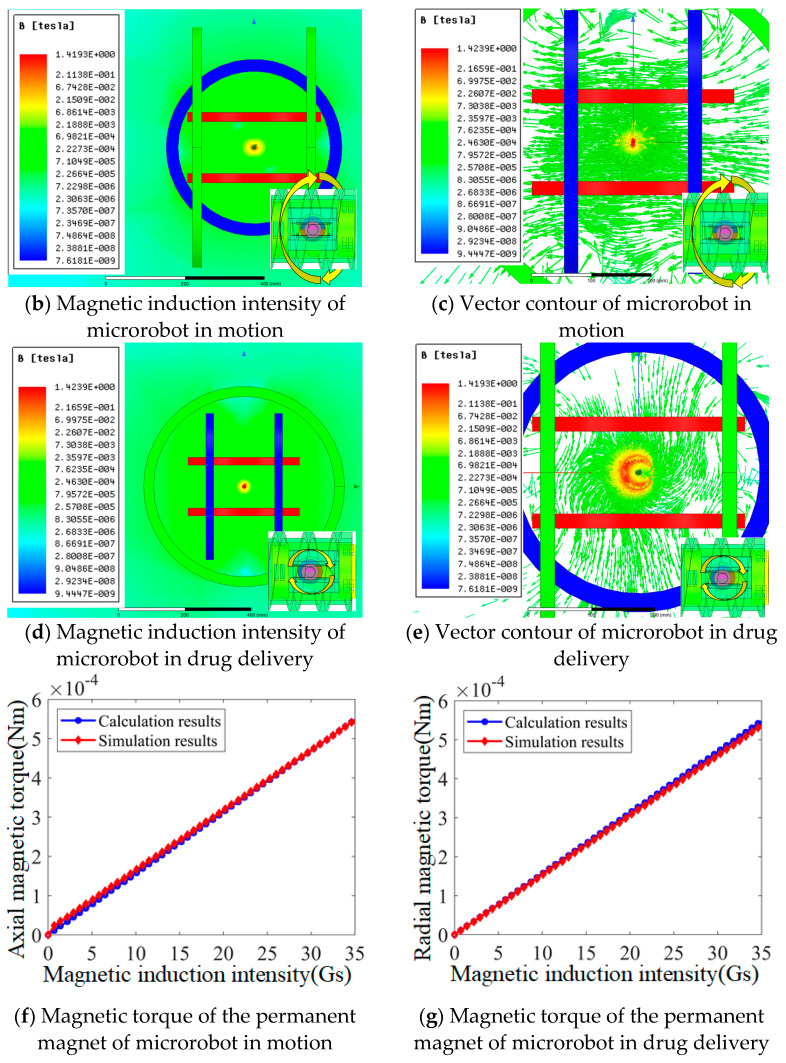
Simulation model and electromagnetic analysis results.

In order to obtain the feasibility of a permanent magnet inside the targeted drug delivery microrobot to realize the two functions of motion and targeted drug application, according to the characteristics of the three-axis Helmholtz coil, a model the same as the experimental platform and the same model as the permanent magnet along the *X* axis embedded in the targeted drug delivery microrobot are established by using electromagnetic simulation software, as shown in Figure 6a.

When the targeted drug delivery microrobot moves, the embedded permanent magnet rotates axially; the magnetic induction intensity contour is shown in Figure 6b, and the vector contour of magnetic induction intensity is shown in Figure 6c. It can be observed that the permanent magnet magnetized radially along the *X* axis is in a uniform magnetic field, and the slip angle between the uniform magnetic field and the permanent magnet is 90°. When the targeted drug delivery microrobot needs to perform the targeted drug application function, the embedded permanent magnet rotates radially, and the magnetic induction contour and vector contour are shown in Figure 6d,e, respectively. It can be observed that the slip angle between the permanent magnet magnetized radially along the *X* axis and the uniform magnetic field is 90°. By changing the magnetic induction intensity of the magnetic field, the radial and axial rotation torques of the permanent magnet are obtained, respectively. The comparison results with the calculation results of Equation (12) are shown in Figure 6f,g. The calculated results are in good agreement with the simulation results. The magnetic torque can realize the axial rotation of the permanent magnet to provide power for the rotation of the targeted drug delivery microrobot, and can also realize the function of radial rotation to promote the drug bin to complete the targeted drug application.

### 4.3. Hydrodynamics Analysis of Targeted Drug Delivery Process

The mesh model of the targeted drug delivery microrobot is divided into two parts: the targeted drug delivery microrobot model and the GI model. The mesh size of the static flow field of the GI model is slightly larger, and the mesh size of the dynamic flow field of the microrobot is relatively small, to balance the accuracy and efficiency of the simulation when the microrobot moves in the GI [25]. In order to ensure the accuracy of the calculation results and prevent large error when the microrobot approaches the inlet or outlet of the GI, the microrobot model is placed in the middle of the GI.

In order to establish the hydrodynamic model, firstly, without considering the intestinal deformation, the central axis of the microrobot coincides with the central axis of the GI. Secondly, taking water as the fluid in the simulation, it is regarded as a viscous incompressible fluid.

In order to obtain more accurate results in the numerical calculation, the solution method is set as the turbulence k-epsilon model, which has wide applicability, high reliability and good convergence, and is especially suitable for external flow problems with complex geometry such as the GI in this simulation [26]. Since the fluid is incompressible, the Couple algorithm is used to solve the coupling equation of pressure and velocity in the simulation.

**Figure 7:**
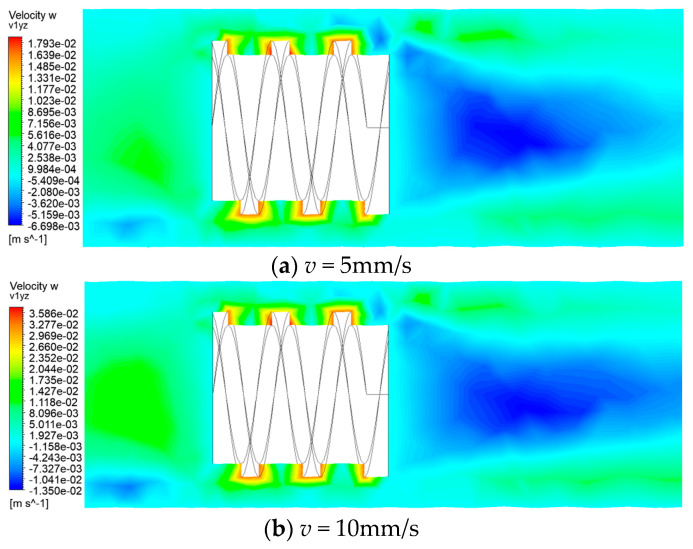

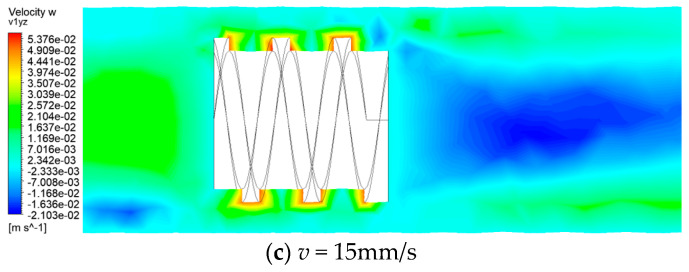
Simulation results for the microrobot motion.

This section only analyzes the motion characteristics of the targeted drug delivery microrobot in the container filled with liquid from the perspective of dynamics. Using finite element analysis software, a model whose model parameters are the structural parameters of the actual targeted drug delivery microrobot is established. Firstly, the motion process of the targeted drug delivery microrobot is analyzed, and the motion characteristics of the targeted drug delivery microrobot when moving are obtained. In Figure 7, the motion of the targeted drug delivery microrobot at the motion speeds of 5 mm/s, 10 mm/s and 15 mm/s is shown, respectively. The results show that the liquid at the tail may not be sprayed evenly, resulting in random shaking and intestinal injury of the microrobot when the speed of the microrobot is low.

**Figure 8:**
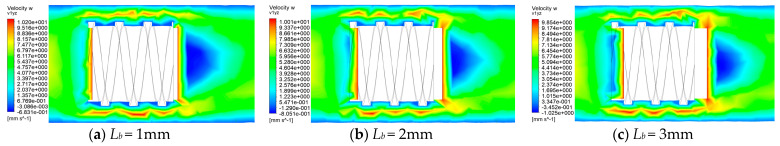
Simulation results of the pushing process of the targeted drug delivery microrobot.

Figure 8 shows the results of a 1 mm, 2 mm and 3 mm length of the drug bin at the speed of 10 mm/s when the targeted drug delivery microrobot reaches the affected area and performs the drug application task. The results show that when the right-hand drug bin performs the drug application task, the fluid velocity close to the drug bin changes the fastest, and the resistance to the fluid increases with the extended length. In other words, if more drugs need to be applied, there will be greater resistance.

**Figure 9:**
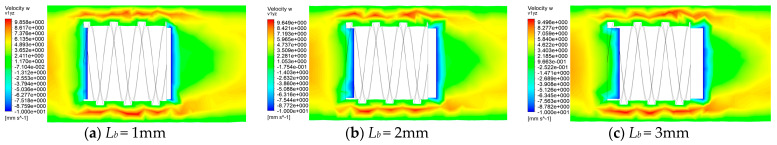
Simulation results of the recovering process of the targeted drug delivery microrobot.

Figure 9 shows the results when the drug release on the right-hand side of the targeted drug delivery microrobot is completed and the left-hand drug bin needs to carry out the drug application task; the right-hand drug bin retracts by 1 mm, 2 mm and 3 mm, respectively, at the speed of 10 mm/s, while the left-hand drug bin extends by 1 mm, 2 mm and 3 mm, respectively, at the speed of 10 mm/s. The results show that the fluid velocity close to the drug bin changes the fastest, which is different from that of the right-hand drug bin. Currently, not only does the push out of the left-hand drug bin need to be subject to the resistance of the fluid, but also the withdrawal of the right-hand drug bin needs to be subject to the resistance. In other words, after the drug application task is completed on one side, the resistance of the microrobot on the other side is greater than that on the last application.

**Figure 10:**
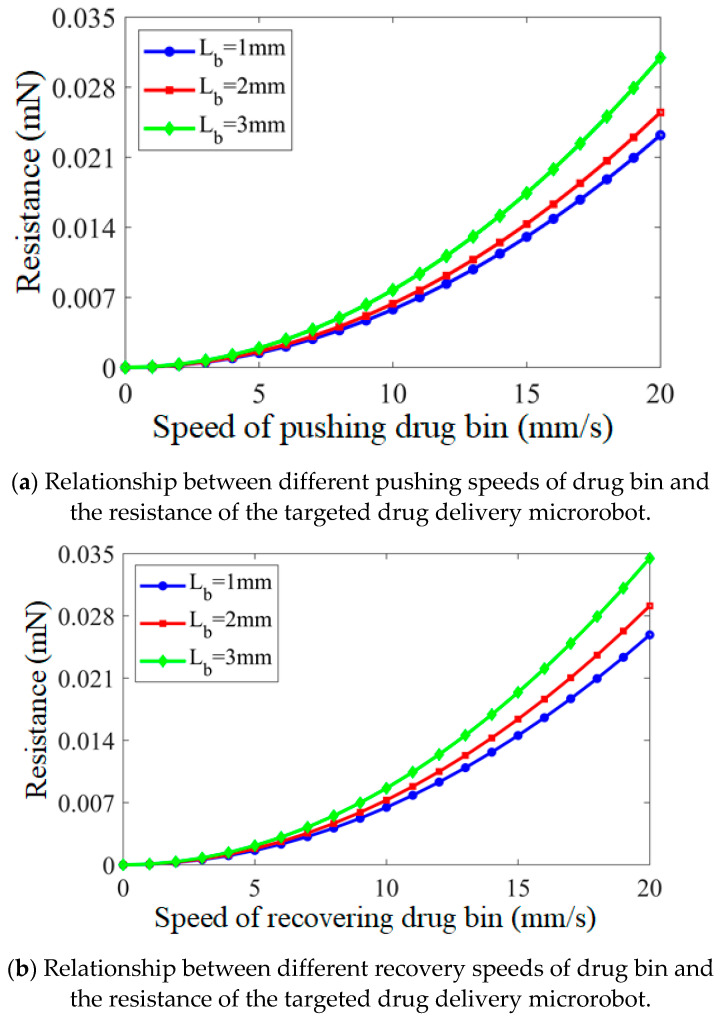
Relationship between the pushing and recovery speed of drug bin and the resistance of the targeted drug delivery microrobot.

Through hydrodynamics simulation, it is simulated that, in the process of drug application, the targeted drug delivery microrobot pushes out the drug bin with different lengths at different speeds. The speed range of pushing out the drug bin is 0–20 mm/s, the increment is 1 mm/s, and the length of pushing out the drug bin is 1 mm, 2 mm and 3 mm, respectively. The different pushing speeds and the resistance of the targeted drug delivery microrobot are shown in Figure 10a. With the increase in the speed of pushing out the medicine bin, the resistance also increases exponentially. In other words, if the drug bin needs to be pushed out at a very fast speed, the external magnetic field needs to control the permanent magnet to increase the magnetic induction strength to a sufficiently high value when it rotates.

When the targeted drug delivery microrobot recovers the drug bin with different lengths at different speeds, the speed range of recovering the drug bin is set as 0–20 mm/s, the increment as 1 mm/s and the length of retracting the drug silos as 1 mm, 2 mm and 3 mm. The different recovery speeds and the resistance force suffered by the targeted drug delivery microrobot are shown in Figure 10b. With the increase in the speed of recovering the drug bin, the resistance also increases exponentially, and the resistance of recovery is greater than the resistance of pushing out, which is because the fluid produces friction on the pushed drug bin. In other words, when the targeted drug delivery microrobot recovers the medicine bin, it needs greater magnetic induction intensity to produce greater thrust to overcome the resistance of the drug bin.

## 5. Experimental Results

**Figure 11:**
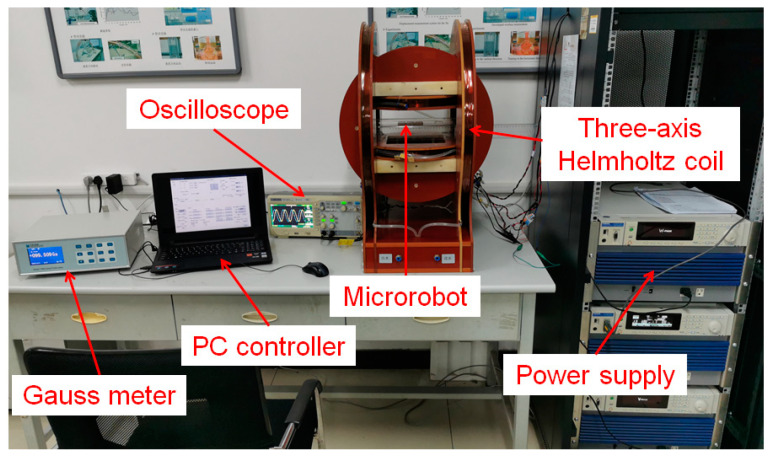
Magnetic actuated capsule microrobotic system.

**Figure 12:**
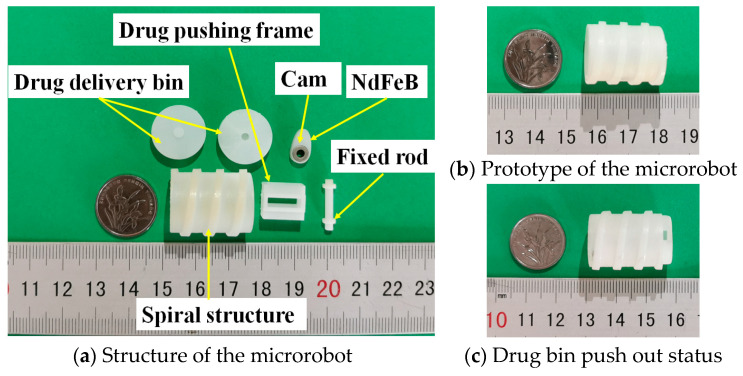
Structure and prototype of targeted drug delivery microrobot.

MACMS is used to test the motion and targeted drug application performance of targeted drug delivery microrobot, as shown in Figure 11. In addition, the prototype of the targeted delivery microrobot is shown in Figure 12. The targeted drug delivery microrobot is placed in the region of the uniform rotating magnetic field generated by the three-axis Helmholtz coil during all experiments [27]. When the signal is sent to the slave treatment system through the master control system, the three-axis Helmholtz coil will produce a uniform universal space rotating magnetic field to provide energy for the targeted drug delivery microrobot. The increment of magnetic field frequency is 1 Hz, which can be adjusted from 0 Hz to 99 Hz, and the direction of the rotating magnetic field can also be adjusted to control the rotation speed and movement direction of the targeted drug delivery microrobot in the fluid-filled pipeline.

### 5.1. Motion Characteristics of Microrobot

**Figure 13:**
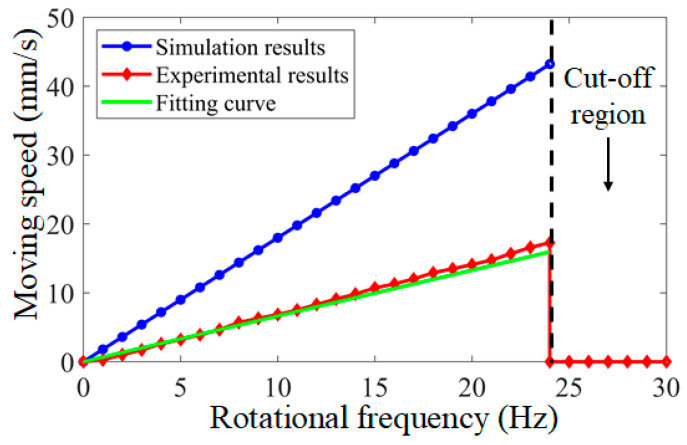
Relationship between rotational frequency and moving speed of targeted drug delivery microrobot.

Firstly, the motion performance of the targeted drug delivery microrobot was evaluated. The test was carried out in a plexiglass tube filled with water. Under the magnetic induction intensity of 6.5 Gs, the relationship between the rotational frequency and moving speed of the targeted drug delivery microrobot could be measured, and the relationship between the rotation frequency and moving speed of the targeted drug delivery microrobot was collected through the hydrodynamic simulation software. The simulation results and experimental results of the motion characteristic of the targeted drug delivery microrobot are shown in Figure 13, which shows that there was a linear relationship between the rotational frequency and moving speed of the microrobot. Due to the errors in the geometric dimensions of the microrobot and pipeline in the experiment, the microrobot could not be consistent with the simulation results.

In addition, the rotation frequency of the external magnetic field was too high when the rotation frequency of the microrobot reached 24 Hz; the rotation frequency of the permanent magnet inside the microrobot could not be consistent with the external magnetic field, and the microrobot reached the cut-off state and could not continue to move [27,28]. Therefore, when the rotational frequency was greater than 24 Hz, this was the cut-off area of the targeted drug delivery microrobot. In addition, the targeted drug delivery microrobot had the same motion performance when moving in the reverse direction.

### 5.2. Drug Delivery Conditions of Microrobot

**Figure 14:**
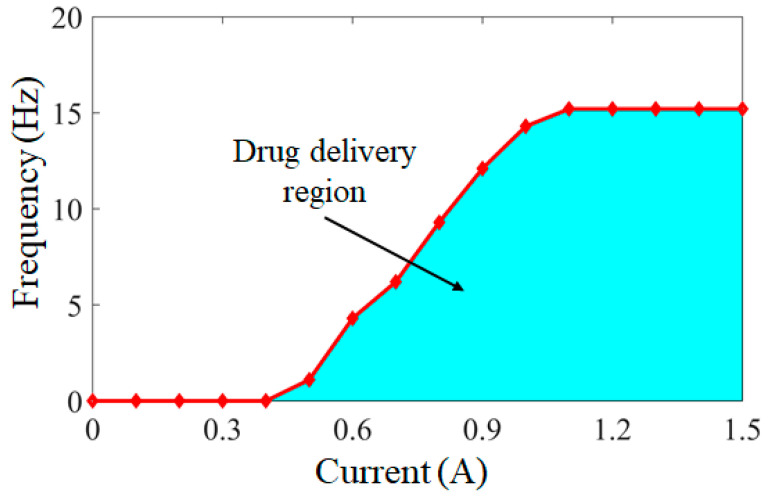
Relationship between the drug delivery region of targeted drug delivery microrobot and the current and frequency of the external magnetic field.

Through the control of the external magnetic field, the conditions under which the targeted drug delivery microrobot can carry out the drug delivery task were simulated. The relationship between the drug delivery region of the targeted drug delivery microrobot and the external magnetic field current and frequency is shown in Figure 14. By adjusting the current of the external coil to 0–1.5 A, the increment is 0.1 A, and the generated magnetic induction intensity is 0–10 Gs. Through 15 groups of experiments, each group was carried out ten times, and the frequency results were taken as the average value. The frequency range of the permanent magnet capable of completing the delivery task is 0–15 Hz. The blue area in Figure 14 is the region capable of completing the delivery task. Any current and frequency in this region can enable the targeted delivery microrobot to carry out the delivery task. When the current is less than 0.4 A, due to the pressure of the external fluid, the thrust of the permanent magnet driven by the magnetic field cannot push out the drug bin. When the current reaches 1.1 A, the maximum frequency that can be applied is 15 Hz. In addition, when the frequency is greater than 15 Hz, the permanent magnet cannot follow the magnetic field frequency and enter the cut-off state due to its own inherent properties, so it cannot complete the drug delivery task. Therefore, according to the specific situation of the drug delivery task, the drug delivery task of the targeted drug delivery microrobot can be completed by controlling in the drug delivery region.

### 5.3. Microrobot Performs Drug Delivery Task

**Figure 15:**
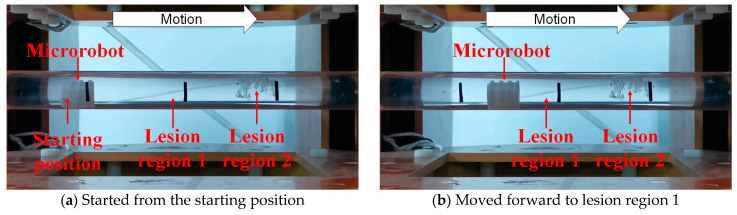

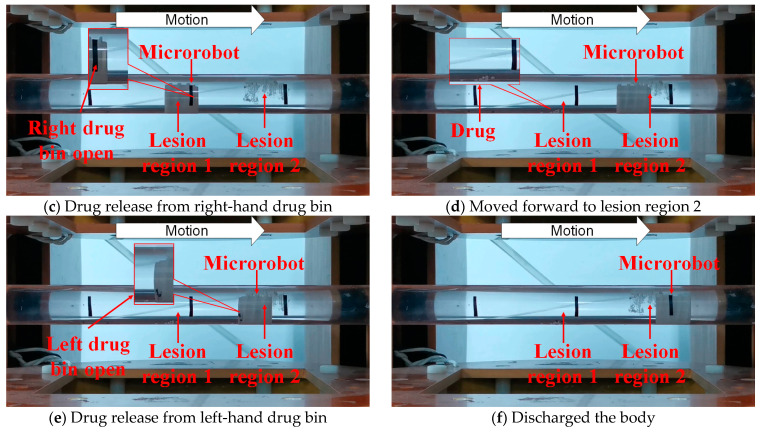
Targeted drug delivery microrobot performs targeted drug application tasks.

**Figure 16:**
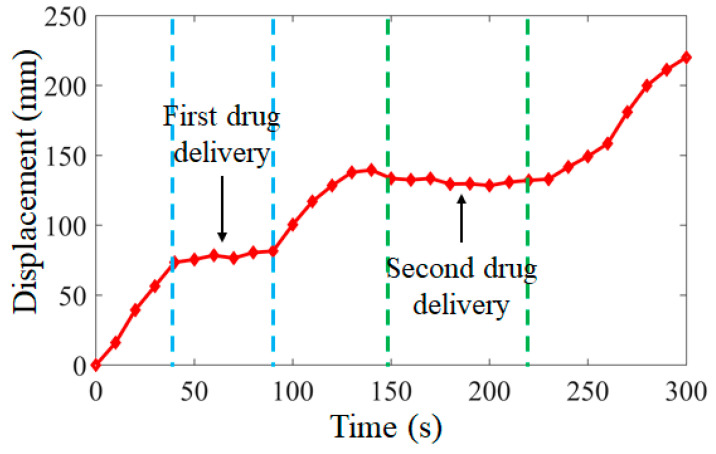
Displacement of targeted drug delivery microrobot during targeted drug application tasks.

According to the task requirements of the targeted drug delivery microrobot in the human GI tract, the complete process of the targeted drug application task was simulated, as shown in Figure 15. The left-hand drug bin of the microrobot was equipped with black granular drugs, and the right-hand drug bin was equipped with white granular drugs. Two lesion regions, 1 and 2, were set for the whole process. The targeted drug delivery microrobot started from the starting position, as shown in Figure 15a. By controlling the external magnetic field, the permanent magnet embedded in the microrobot rotated axially and moved forward by the reaction force of the fluid, as shown in Figure 15b. After reaching lesion region 1, the permanent magnet inside the targeted drug delivery microrobot rotated radially by controlling the external magnetic field, driving the cam structure to push the drug bin on the right to release the drug, as shown in Figure 15c. After the drug treatment of lesion region 1, the microrobot continued to move forward, and the granular drug at lesion region 1 could be observed, as shown in Figure 15d. When the targeted drug delivery microrobot reached lesion region 2, the external magnetic field was controlled to make the cam structure push the left-hand drug bin and release the drug, as shown in Figure 15e. Finally, the targeted drug delivery microrobot completed the task and discharged the body, as shown in Figure 15f. The displacement results of the targeted drug delivery microrobot are shown in Figure 16, and the whole process took 300 s.

## 6. Conclusions

In order to realize the function of targeted drug delivery for human GI diseases in a limited space, the characteristics of a targeted drug delivery microrobot are studied in detail in this paper. By controlling the magnetic field generated by the three-axis Helmholtz coil, the rotation, operation and targeted drug delivery of the targeted drug delivery microrobot can be accurately controlled. The driving source of the cam structure inside the targeted drug delivery microrobot is a radially magnetized permanent magnet. The forward, backward and moving speed of the microrobot can be realized when the permanent magnet rotates axially through the fixed rod. By fixing with the cam, the drug release from the drug bin can be controlled when the permanent magnet rotates radially, to achieve multiple targets and control the amount of drug for drug application. Through the drug application experiment simulating the human intestinal tract, it is verified that the targeted drug delivery microrobot can realize the two functions of motion and drug application of the microrobot by an internal single permanent magnet. In the future, we will further study the precise dosage of the targeted drug delivery microrobot, treat specific clinical diseases and conduct further research in the real environment of the animal intestine.

## Figures and Tables

**Table 1:** Specifications of the targeted drug delivery microrobot.

Microrobot	Parameter
Diameter of microrobot (mm)	9.5
Length of microrobot (mm)	24
Weight of microrobot (g)	4.0
Thickness of spiral structure (mm)	2
Pitch of spiral structure (mm)	6.6
Height of spiral structure (mm)	1
Material of body	Photosensitive resin
Size of permanent magnet (mm × mm × mm)	6 × 3 × 4
Weight of permanent magnet (g)	1.0
